# Development and Testing of an Automated 4-Day Text Messaging Guidance as an Aid for Improving Colonoscopy Preparation

**DOI:** 10.2196/mhealth.5289

**Published:** 2016-06-21

**Authors:** Benjamin Michael Walter, Peter Klare, Bruno Neu, Roland M Schmid, Stefan von Delius

**Affiliations:** ^1^II. Medizinische Klinik und PoliklinikKlinikum rechts der IsarTU MünchenMünchenGermany

**Keywords:** short message service, patient education, colonoscopy, colonoscopy preparation

## Abstract

**Background:**

In gastroenterology a sufficient colon cleansing improves adenoma detection rate and prevents the need for preterm repeat colonoscopies due to invalid preparation. It has been shown that patient education is of major importance for improvement of colon cleansing.

**Objective:**

Objective of this study was to assess the function of an automated text messaging (short message service, SMS)–supported colonoscopy preparation starting 4 days before colonoscopy appointment.

**Methods:**

After preevaluation to assess mobile phone usage in the patient population for relevance of this approach, a Web-based, automated SMS text messaging system was developed, following which a single-center feasibility study at a tertiary care center was performed. Patients scheduled for outpatient colonoscopy were invited to participate. Patients enrolled in the study group received automated information about dietary recommendations and bowel cleansing during colonoscopy preparation. Data of outpatient colonoscopies with regular preparation procedure were used for pair matching and served as control. Primary end point was feasibility of SMS text messaging support in colonoscopy preparation assessed as stable and satisfactory function of the system. Secondary end points were quality of bowel preparation according to the Boston Bowel Preparation Scale (BBPS) and patient satisfaction with SMS text messaging–provided information assessed by a questionnaire.

**Results:**

Web-based SMS text messaging–supported colonoscopy preparation was successful and feasible in 19 of 20 patients. Mean (standard error of the mean, SEM) total BBPS score was slightly higher in the SMS group than in the control group (7.3, SEM 0.3 vs 6.4, SEM 0.2) and for each colonic region (left, transverse, and right colon). Patient satisfaction regarding SMS text messaging–based information was high.

**Conclusions:**

Using SMS for colonoscopy preparation with 4 days’ guidance including dietary recommendation is a new approach to improve colonoscopy preparation. Quality of colonoscopy preparation was sufficient and patients were highly satisfied with the system during colonoscopy preparation.

## Introduction

An optimal bowel preparation for colonoscopy is one of the most important cornerstones for gastroenterologists. A poor bowel preparation is associated with decreased adenoma detection, longer examination, and increased costs by virtue of the decreased interval to repeat examination [1-3]. Bowel preparation is inadequate in an estimated 15% to more than 20% of patients undergoing colonoscopy [2,4]. Misunderstanding dietary recommendations and cleansing instructions, as well as noncompliance, plays a major role in poor bowel preparation [4]. Information about diet and the preparation procedure is usually provided to patients before endoscopy after scheduling the colonoscopy appointment. Further education and continuous guidance of patients before colonoscopy has been shown to ensure quality of colonoscopy preparation and patient compliance [5]. In Germany, colon cancer prevention program starts at the age of 50 years, with a full insurance-covered outpatient colonoscopy starting at the age of 55 years. Participation rate in colon cancer prevention is low in Germany as it is in the rest of Europe. Colonoscopy preparation is especially reported to be unpleasant [1].

Over the last few years, there has been an enormous increase in the use of mobile phones in the overall population as well as among patients of all ages. Integration of such new media into colonoscopy preparation could help optimize the preparation procedure. We decided to use the well-established medium short message service (SMS), because SMS text messaging can be easily used for every type and age of mobile phone.

First, we performed a preevaluation to assess mobile phone usage in our patient population. A questionnaire was administered in order to analyze how many people already own and use mobile phones.

Second, an automated SMS text messaging system for colonoscopy preparation starting 4 days before colonoscopy was developed and tested in the following study.

The primary aim of the *PERICLES I* (*prospective evaluation for improvement of colonoscopy preparation procedure by software supported visualization*) study was to evaluate if a newly developed automated SMS text messaging reminder system starting 4 days before colonoscopy, containing dietary and behavioral recommendations, is feasible. Secondary end points were patient satisfaction with the system and the quality of bowel preparation.

## Methods

To assess mobile phone usage in our patient population, we performed a preevaluation by administering a questionnaire.

### Preevaluation of Mobile Phone Usage

For the analysis of percentage of patients who own and regularly use a mobile phone, a questionnaire study was initiated at our hospital. In total 349 patients were invited to participate. A total of 300 patients agreed to participate. Age, sex, and additional information about the type of mobile phone used—that is, mobile phone, smartphone (eg, iPhone, Samsung), or none—were collected and analyzed.

### Text Messaging System Development

In cooperation with the company SmartPatient a fully automated, Web-based SMS text messaging reminder system containing important information on colonoscopy preparation was developed. It contains 15 messages (up to 160 characters each) that could be sent to a patient's mobile phone. For the content of the SMS text messages we decided to follow the general recommendations for outpatient colonoscopy, provided in a paper-based leaflet, used at our hospital starting 4 days before colonoscopy.

The program sends Web-based SMS text messages, adjusted to the specific date and time of colonoscopy appointment. Its guidance covers the patient starting 4 days before colonoscopy with behavioral and dietary recommendations. At the specified time, the patient is reminded to start laxative intake and consume recommended amount of clear fluids.

### Text Message Contents

**Figure 1:**
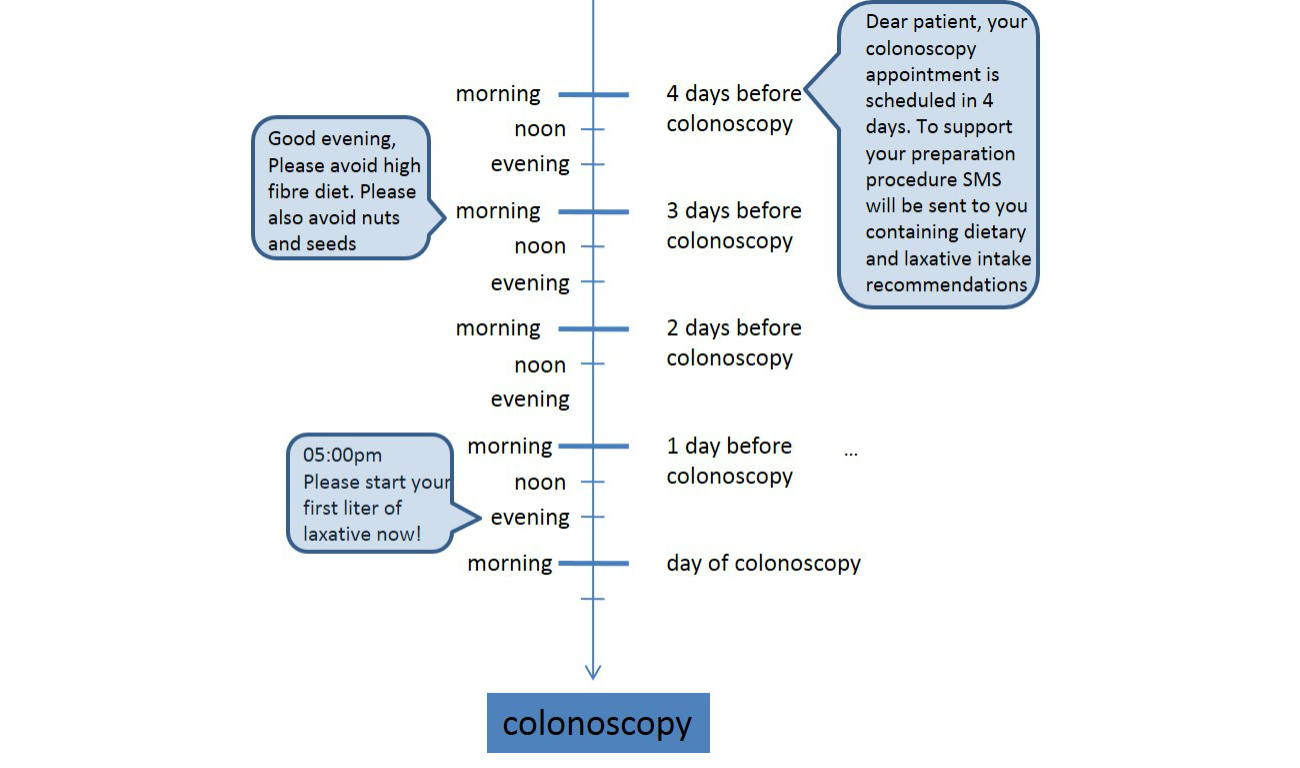
Graphical workflow of the text messaging (short message service, SMS)-guided colonoscopy preparation; examples of SMS text messages.

As characters for SMS text messages are limited, some text messages are split into two. First, second, and third messages contain a welcome message and general information about colonoscopy preparation and safety advices (eg, car driving is prohibited after colonoscopy with sedation). Dietary information is provided for each day starting with the fourth SMS text message 3 days before colonoscopy. On the day before colonoscopy the patients receive in total 6 SMS text messages containing information about starting and continuing of laxative intake and dietary recommendations. The last SMS text message is automatically sent 1 hour before the colonoscopy appointment. For graphical scheme of the SMS text messaging reminder system, please see Figure 1.

After developing the SMS text messaging system, a prospective study was conducted at the II. Medizinische Klinik, Klinikum rechts der Isar, Technische Universität München, Munich. The study protocol was approved by the local ethics committee. Informed consent was obtained from the study participants.

### Colonoscopy Preparation Scheme

At our institution, colonoscopy preparation standard for all patients is a regular polyethylene glycol (PEG)–based split-dose regimen (MOVIPREP; Norgine, England). Explanations of the regular colonoscopy preparation procedure are given during informed consent discussion several days before endoscopy by the endoscopist. Furthermore, a leaflet containing detailed diet and preparation recommendations is given to every patient before colonoscopy. For analysis of quality assurance, colonoscopy preparation is routinely measured by the Boston Bowel Preparation Scale (BBPS) [6]. The BBPS was developed to limit interobserver variability in the rating of bowel preparation quality, while preserving the ability to distinguish various degrees of bowel cleanliness. A 4-point scoring system is applied to the 3 broad regions of the colon: the right colon including the cecum and ascending colon, the transverse colon including the hepatic and splenic flexures, and the left colon including the descending colon and rectum. Every segment receives a segment score from 0 to 3 (0, minimum cleanliness to 3, maximum cleanliness). These segment scores are summed for a total BBPS score with a possible achievable total count of 9 points (3 for each colon region: left colon, transverse colon, and right colon). The BBPS reflects a better discrimination in colon regions and quality of colon preparation in comparison with other preparation scales. Previous studies have shown that a BBPS score of 5 is an important clinical threshold [6,7].

### Feasibility Study Performance

A total number of 20 patients undergoing outpatient colonoscopy were included in the SMS text messaging–supported colonoscopy preparation group from November 2013 to January 2014. Inclusion criteria were outpatient colonoscopy, written informed consent, age >18 years, and mobile phone with a SIM (subscriber identity module) card of a German (national) mobile phone provider. Exclusion criteria were phenprocoumon therapy, diabetes mellitus with insulin therapy, pregnancy, recent neurologic illnesses, and reported electrolyte disturbances.

Date and time of colonoscopy and mobile phone numbers of the SMS study group participants were collected and entered into a fully automated SMS text messaging reminder system (SmartPatient GmbH, Munich, Germany). Participants of the SMS study group additionally received the aforementioned SMS text messages. The information contained was in accordance with the colonoscopy preparation leaflet, which was handed to the patient in advance. No additional contents were provided by the short messages (Figure 1).

For safety reasons the study participants were informed to follow the instructions on the provided leaflet in case of delayed or missing SMS text messages or unclear information about the preparation steps.

A questionnaire was given to the SMS study group participants, containing the following topics to be evaluated by a numeric rating scale (NRS) or yes/no answers: (1) history of prior colonoscopies (experience in bowel preparation), (2) SMS text message received or not, (3) extent of discomfort caused by the colonoscopy preparation procedure, (4) whether the information provided by SMS text message was helpful, (5) whether information provided by SMS text message was inhibitory toward preparation, (6) whether the patient would favor the use of SMS text messaging–supported colonoscopy preparation again, and (7) whether patients would recommend the SMS text messaging reminder system to friends or family members undergoing colonoscopy.

Satisfaction with the SMS text messaging system was assessed using an NRS from 1 to 10 (1, not helpful to 10, very helpful; 1, not inhibitory to 10, very inhibitory).

For reasons of comparison, BBPS data of 20 patients who underwent outpatient colonoscopy at our institution during the study period, matching in age, sex, and indication for colonoscopy, were compared with the study data.

The primary end point was the feasibility of the automated SMS text messaging reminder system for colonoscopy preparation assessed as stable function of the system, technical success, and consecutive feasibility of colonoscopy preparation and colonoscopy.

Secondary end points included patient satisfaction (perception of the message contents as helpful or as a hindrance, whether the reminder system would be chosen for next colonoscopy again, and whether the SMS text messaging system could be recommended to friends and relatives) and quality of bowel preparation assessed by the BBPS as rated by the endoscopist. We defined a threshold of a BBPS score of 5 or higher for a sufficient bowel preparation for colonoscopy [6,7].

### Statistical Analysis

A total of 20 patients were planned to be included in the feasibility study. Descriptive statistics were computed for all variables to provide means and standard deviations (SDs) for continuous variables and frequencies for categorical variables. Total BBPS scores were calculated (SMS study group and controls). *P* values correspond to Mann-Whitney *U* test. The results for colon preparation were dichotomized to adequate preparation (BBPS total score 5-9) and inadequate colon preparation (BBPS total score <5). All statistical analyses were performed with statistical software GraphPad Prism (GraphPad Software Inc, La Jolla, CA, USA).

## Results

### Preevaluation of Mobile Phone Usage

For the questionnaire study 300 patients were analyzed. Mean age was 61.4 (standard error of the mean, SEM 18.5) years. There were 133 female participants and 167 male participants (male to female ratio was 1.3:1). In total there were 119 patients with smartphones (39%), 128 with mobile phones (43%), and 53 patients (18%) without a mobile phone. Patient characteristics and results are presented in Table 1.

**Table 1:** Patient characteristics of mobile phone usage.

Characteristic	Mobile phone	Smartphone	None
Number of patients, n (%)	128 (43)	119 (39)	53 (18)
Sex (male/female)	64/64	76/43	27/26
Age in years, mean (SEM^a^)	65.8 (12.9)	47.3 (15.5)	82.2 (8.6)

^a^ SEM: standard error of the mean.

### Text Messaging System Feasibility Study

#### Patient Characteristics

For the study a total of 20 patients who got an appointment for outpatient colonoscopy were included. Male to female ratio was 1:1 (10 males, 10 females). Data from outpatient colonoscopies were collected as control (Table 2). Controls were taken from the colonoscopy database of the hospital. Outpatient colonoscopies performed during the recruiting period of the study were included. Matching criteria were as follows: age (±1 year), sex, first or previous colonoscopies (in our hospital), and preparation with PEG (prescription). In case of several matching patients, data of the patient with the highest BBPS result were taken from the database to avoid further bias.

#### Primary End Point

The text messages were received by 19 of 20 participants (Table 2). For 1 participant an invalid SIM card by mobile phone provider caused a 20-minute delay in every SMS text message delivered.

#### Secondary End Points

**Figure 2:**
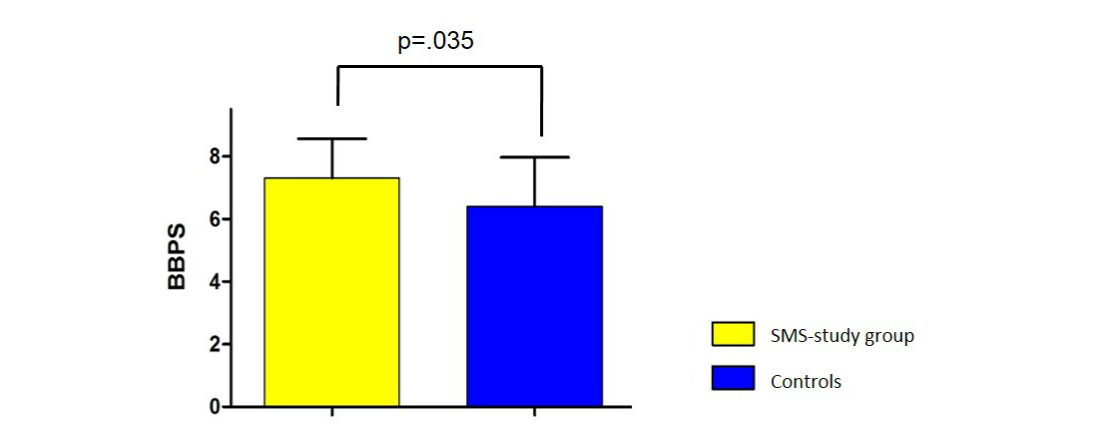
Average Boston Bowel Preparation Scale (BBPS) score in the short message service (SMS) study group was significantly higher in comparison with the control group (7.3 vs 6.4, *P*=.035). BBPS: 0, minimum to 9, maximum.

**Figure 3:**
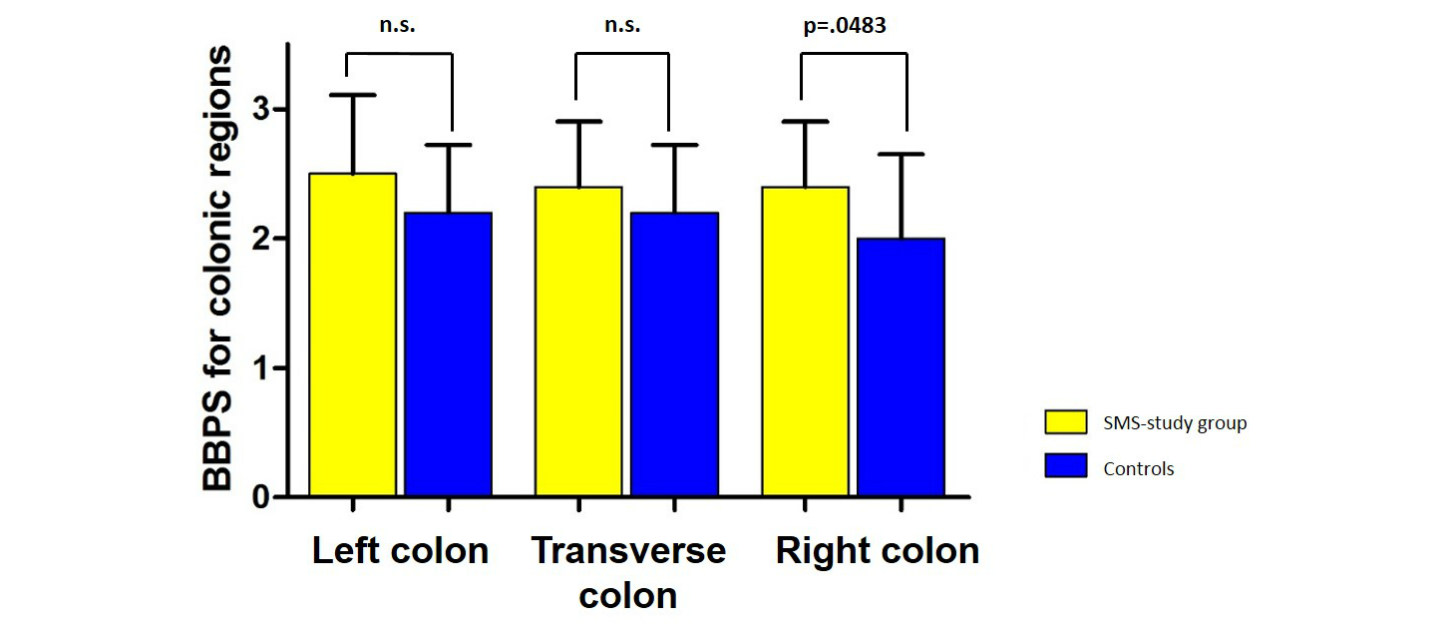
The average Boston Bowel Preparation Scale (BBPS) score (0, minimum to 3, maximum) split for each colon region. SMS: short message service; n.s.: not significant.

No total BBPS score lower than 5 points was recorded in the SMS study group, whereas 1 patient had a BBPS score of <5 in the control group. Mean BBPS score of the SMS study group was 7.3 (SEM 0.28) in comparison with mean 6.4 (SEM 0.35) in the control group, which is a significant improvement, calculated by Mann-Whitney *U* test (*P*=.035 for difference; Figure 2). Regarding the left, transverse, and right colon regions, there was a certain improvement in the BBPS score of all colon regions (Figure 3). The mean BBPS score of the SMS study group for the left colon was 2.5 (SEM 0.13), which was higher in comparison with mean 2.2 (SEM 0.12) in the control group; however, the improvement was not statistically significant (*P*=.0816 for difference; Figure 3). The mean BBPS score of the SMS group for the transverse colon was 2.4 (SEM 0.11), which was higher in comparison with mean 2.1 (SEM 0.11) in the control group; however, the improvement was not statistically significant (*P*=.2482 for difference; Figure 3).The mean BBPS score of the SMS group for the right colon was 2.4 (SEM 0.11), higher in comparison with mean 2.0 (SEM 0.14) in the control group, which is a significant improvement (*P*=.0483 for difference; Figure 3).

All study participants of the SMS group stated they would use the SMS text messaging reminder system again. Of 20 participants, 19 stated they would recommend the system to their friends and relatives, whereas 1 participant was willing to recommend the SMS text messaging system only if it was a step toward a colonoscopy preparation app for smartphones. When asked if the reminder system was helpful to get the colonoscopy preparation done, patients reported an average NRS score for usefulness of 7.8 (SD 2.2, n=18). On the contrary, the SMS text messaging reminder system was not found to be inhibitory by an average NRS score for inhibitory effect of the text message of 1.1 (SD 0.31, n=19; Table 2).

**Table 2:** Patient characteristics.

Characteristic	SMS group	Control group
No. of patients	20	20
Sex (male/female)	10/10 (50%/50%)	10/10 (50%/50%)
Age in years, mean (SD^a^)	46.5 (12.6)	46.5 (13.0)
Method of bowel preparation, PEG^b^ solution no. (%)	20 (100.0)	20 (100.0)
First colonoscopy (yes/no)	13/7 (65%/35%)	12/8 (60%/40%)
SMS^c^ received and followed instructions (yes/no)	19/1 (95%/5%)	N/A^d^
Preparation procedure is perceived as stressful^e^, mean (SD^a^)	5.6 (2.4)	N/A
SMS information perceived as helpful information^f^, mean (SD)	7.8 (2.2)	N/A
SMS information perceived as hindrance^g^, mean (SD)	1.1 (0.31)	N/A
Reuse of SMS reminder system for another colonoscopy (yes/no)	20/0 (100%/0%)	N/A
Recommendation of SMS system to friends and relatives (yes/no)	19/1 (95%/5%)	N/A

^a^ SD: standard deviation.

^b^ PEG: polyethylene glycol.

^c^ SMS: short message service.

^d^ N/A: not assessed.

^e^ Evaluation: 1, no stress to 10, very stressful.

^f^ Evaluation: 1, not helpful to 10, very helpful.

^g^ Evaluation: 1, not a hindrance to 10, great hindrance.

## Discussion

Adequate bowel preparation is a prerequisite for an effective colonoscopy. Inadequate bowel preparation occurs in 15% to more than 20% of all examinations [1,2]. It has been reported that many patients have problems in handling the laxative and following dietary recommendations before the colonoscopy appointment [8-12]. Patient compliance is generally reduced in case of unclear instructions or uncertainty in therapy recommendations. These data stress the importance of proper patient education [5]. The approach of using new media that are spreading among the population is still poorly examined. We now report the development and testing of an automated Web-based SMS text messaging system to guide the patient through colonoscopy preparation.

### Preevaluation to Assess Mobile Phone Usage

Preevaluation performed to assess mobile phone usage in our patient population revealed that the majority of our patients are already equipped with mobile phones and use them in everyday life. Therefore the approach to include new media such as SMS text messaging in colonoscopy preparation could be quite reasonable. The mean age of patients with a mobile phone, in comparison with smartphone users, was much higher; therefore we arrived at the conclusion that the population undergoing screening colonoscopy is still better supported by a mobile phone (SMS)–derived solution.

### Text Messaging–System Development and Feasibility Study

This study showed that an automated, Web-based SMS text messaging reminder system is feasible for outpatients. Technical success (SMS text messages were sent and received in time, the Web-based SMS text messaging system functioned satisfactorily, no remarkable bugs had to be fixed during the study) was achieved in 19 of 20 patients. In the case of 1 patient a technical problem was caused by an invalid SIM card from the mobile phone provider. There was a 20-minute delay in delivering each SMS text message. Importantly, colonoscopy was performed without problems in all patients of the SMS study group.

Patients perceived the SMS text messaging service as very helpful and stated that they would recommend its use to friends and relatives.

Moreover, we could observe a tendency toward improvement in the quality of the preparation procedure. When compared with control subjects who followed standard verbal instructions and a leaflet for bowel preparation, the SMS study group participants achieved better, but statistically significant, results of bowel cleansing. This was reflected by a higher BBPS count. A BBPS score of <5 is generally considered to be an insufficient preparation result, which leads to the recommendation to repeat the examination [6,7]. In comparison with the control group, none of the SMS study group participants had a BBPS score of <5.

Several approaches toward improvement of patient guidance and education have been evaluated in the past. A visual aid based on cartoons as well as video education or telephone-based reminder systems improved patient satisfaction and bowel preparation quality [13-16]. All approaches had specific limitations. For example, video-based education is stationary and needs additional equipment (eg, DVD player or television). A telephone-based reminding system or an existing SMS text messaging–based system consists only of a single (manual) phone call or SMS text message the day before the colonoscopy.

A continuous patient guidance and personal contact throughout the days before colonoscopy would be the most favorable situation for improving colonoscopy preparation results. Therefore, we believe that inclusion of dietary and behavioral recommendations instead of only reminding the patient to start laxative intake is necessary.

Nowadays, mobile phones, but not yet smartphones, are widely used even among the older population. Short message service is already an established way of communication. By using an automated SMS text messaging reminder system, a closer guidance and education of the patient can be ensured. This allows repeating the information for the most crucial steps of colonoscopy preparation in a time-adjusted manner. In order to save most convenient usability for the patient, in general different languages are possible to be used in SMS for colonoscopy preparation. Translation of the text messages into foreign languages can even open up higher participation in colon cancer screening.

### Study Limitations

The study is subject to some limitations. As the automated SMS text messaging–aided colonoscopy preparation system was newly developed, the design of the study was a single-center feasibility study with a consecutive, very small number of study participants. The extended exclusion criteria caused a long period of patient recruitment and the results are very preliminary. The real effect of an automated SMS text messaging reminder system on the quality of bowel cleansing has, therefore, to be shown in larger randomized studies. We also understand that statistics are very limited because of the small number of study participants, and therefore conclusions regarding improvement of bowel cleanliness are also very limited.

However, we would like to point out that automated SMS text messaging guidance covering the whole period of colonoscopy preparation could be a new approach to achieve a higher degree of bowel cleanliness.

### Conclusions

In conclusion, an automated SMS text messaging reminder system starting 4 days before colonoscopy appointment, containing dietary recommendations and recommendations for laxative intake, is technically feasible and helpful during colonoscopy preparation. It could help to ensure procedure quality and improvement of patient comfort.

## Abbreviations

BBPS: Boston Bowel Preparation Scale
NRS: numeric rating scale
SD: standard deviation
SEM: standard error of the mean
SIM: subscriber identity module
SMS: short message service
PEG: polyethylene glycol
